# How learner engagement impacts non-formal online learning outcomes through value co-creation: an empirical analysis

**DOI:** 10.1186/s41239-022-00341-x

**Published:** 2022-07-08

**Authors:** Cenlan Wang, Tala Mirzaei, Tao Xu, Hui Lin

**Affiliations:** 1 Unisoc Corporation, Shanghai, 201203 China; 2 Department of Information Systems and Business Analytics, College of Business, Florida International University, Miami, FL 33199 USA; 3 School of Economics and Management, Tongji University, Shanghai, 200092 China

**Keywords:** Learner engagement, Learning outcomes, Personalized value, Platform value, PLS-SEM, Value co-creation

## Abstract

From the perspective of service science and its core concept of value co-creation, promoting learner engagement is critical for learning outcomes in a non-formal online learning environment. To promote online learning performance, we study how multidimensional learner engagement affects both instrumental and experiential learning outcomes. By incorporating the service-dominant logic perspective into the research model, we designed an online survey to investigate the impact of platform value co-creation on learners’ engagement outcomes. By employing a partial least squares-structural equation modeling (PLS-SEM), the results show that behavioral engagement, cognitive engagement, and emotional engagement have a significant impact on learning outcomes through the mediating effect of platform value, a second-order hierarchical latent variable. This study has multiple theoretical contributions and practical implications. First, we found new evidence that pursuing good learning outcomes in a non-formal online learning setting is not just a technological architecture or pedagogic guidelines, but also a “win–win” value co-creation process. Second, our results confirm the posited direct and indirect effects, thus evidencing functional value, emotional value, social value, and personalized value as components of the platform value construct, and it as a driver and mediator for better online learning outcomes. Third, our results underscore the importance of platform value in studying the impact of learner engagement on learning outcomes and provide a sharper theoretical lens to evaluate online learning platform value from the perspective of online learners.

## Introduction

### Bringing service science to the field of online learning

The number of learners using online learning platforms has reached nearly 200 million in recent years (Narang et al., 2021). Especially under the influence of COVID-19, universities in many countries have adopted online education methods to prevent the spread of the epidemic (Lin et al., 2021). In contrast to formal online learning, non-formal online learners have more chances to choose their preferred platforms and have more channels to create value for themselves. A range of digital technologies such as laptops, tablets, and smart phones are embraced by online learners to support their non-formal learning. However, research on online learning, which is treated as a kind of service, is still in its infancy. As stated by Larson (2009) from the Massachusetts Institute of Technology, “Education as a service industry has always been in urgent need of serious study.” With the advancement of technology, Seo et al. (2021) introduced artificial intelligence systems into online learning to meet the interaction between learners and instructors, and provide online service for them. Online learners are online service users, so the background of online learning is service science. Service science is an emerging interdisciplinary research field that focuses on basic science, models, theory and applications, and promotes service innovation, competition, and well-being by co-creating value. Traditionally, learners are more likely to be passive participants in the classroom. However, the nature of online learning has shifted the learning process from traditional teacher-centered to learner-centered, emphasizing active learning rather than passively relying on teachers or instructors. Researches on the engagement of online learners and the impact spontaneously arise. For example, Jung and Lee (2018) employed structural equation modeling to promote learner’s participation and persistence in online learning. Online learning has become a critical context for end-users (Garg et al., 2018). A fundamental problem in service science is how to understand value co-creation phenomena. Nevertheless, in the field of online learning, there are limited studies assuming learner engagement as user engagement, which is the prerequisite for value co-creation process (Storbacka et al., 2016). The process of creating value in most research of online context is rapidly shifting from a company-centered perspective to a personalized customer experience perspective. And given the sparse research examining the individual’s role in value co-creation, scholars have called for research to understand value co-creation phenomenon from the individual’s point of view (Kleinaltenkamp et al., 2017).

### Value co-creation in online learning

Value co-creation allows users to construct personalized and differentiated service experiences together with service providers according to their unique background (Prahalad & Ramaswamy, 2004). Based on service-dominant logic (SDL), instead of providing value, the service provider provides a value proposition. Users can play a variety of roles, including designers, processors, negotiators, payers, quality controllers, maintainers, and even employees. Literature about SDL describes value as co-created through the integration of resources and determined uniquely by the individuals (Vargo & Lusch, 2015). According to Zhang et al. (2017), user engagement has a direct and positive influence on user value creation. User engagement in online learning is learner engagement, which is a vital way for value co-creation.

Value co-creation requires collaborative learning. Without knowledge sharing, there will be no collaborative interaction and no co-creation of value (Stahl & Hesse, 2009). Collaborative interaction requires a shared understanding within the group. Agredo-Delgado et al. (2021) used the results obtained through a specific experimental analysis to verify the feasibility and effectiveness of building shared understanding in problem-solving activities. It also applies to the value co-creation in online learning. At present, learners have already recognized the significance of online learning as a channel for learning, and learners play an active role in the value they presume. Fostering learner engagement is essential to achieve desired learning outcomes (Panigrahi et al., 2018). Engagement is a primary factor influencing the effectiveness and quality of online learning programs (Mu et al., 2019). The spirit of cooperation, task dependence and social interaction all contribute to enhance learner engagement (Sun et al., 2020). Some scholars have also focused on the relationship between gamified environment and learner engagement (Lavour et al., 2021; Zhang & Yu, 2021). Moreover, by encouraging learner engagement, collaboration dimensions are added to online learning, strengthening learner interaction and communication, and enhancing the learner experiences (Mostafa, 2015). From Henrie's review (2015), learners' cognitive engagement has a positive impact on learners' perceived value. Researches have linked behavioral, cognitive, and emotional engagement to learning outcomes, such as achieving desirable academic and social outcomes, and even feeling emotionally connected (Yang & Lau, 2019).

The focus of the present study takes online learning service as a value co-creation process with the input of learner engagement and the output of learning outcomes. Value co-creation, a user-oriented theory, is similar to online learning as a learner-oriented field. However, the causal relationships between the factors influencing value co-creation in academic environments have not been taken into consideration by researchers (Monavvarifard et al., 2019). Few studies have linked learner engagement to learning outcomes from SDL. Additionally, both “value” and “co-creation” are metaphorical in construction. This study provides a more specific description of the value co-creation process by an empirical study.

The rest of this paper is structured as follows. In the next section, we review the theoretical background of this research. Then, we explain our conceptual model and hypotheses. Next, we explain the PLS-SEM (partial least squares-structural equation modeling) methodology and tests our SEM model by empirical data analysis from an online survey. Finally, a discussion, theoretical and managerial implications, conclusions and limitations of this study complete the paper.

## Related literature and theoretical framework

### Value co-creation and SDL

Value co-creation is a process in which enterprises and users create user experiences through interaction of the platform, and value is embedded in users' personalized experiences. SDL gives priority attention to the matter of understanding and delivering positive and unique user experiences because it shapes users’ continuance intention (Wang, 2015). Thus, learner engagement and user experiences are assumed to predict value creation performance and learning outcomes drawing on value co-creation theory and in the light of SDL. For example, in the context of collaborative innovation communities, value co-creation represents the value that is co-created by members through the integration of resources, enabled by interactive technology platforms (Akman et al., 2018). Platform users take both functional and emotional considerations into account when shaping the value co-creation process (Kamboj et al., 2018). Value is co-created through the joint efforts of companies, employees, users, government agencies, and other entities associated with any given exchange, but ultimately determined by the users (Vargo & Lusch, 2018). Unique service experience is crucial because service value is phenomenologically determined, and it is a service experience that connects the customer with the service provider during the service provision process (Vargo & Lusch, 2018).

However, there is still a lack of clarity about different dimensions of users’ value in value co-creation (Xie et al., 2016). Currás-Pérez et al. (2018) proposed three dimensions of user perceived value, shown in Table 1. In addition to these three value dimensions, we also test personalized value as another component of platform value from the perspective of users. From the perspective of SDL, the platform value is the perceived value of the users. When the service meets the users' needs, they have more motivation to value co-creation. In this process of continuous reconfiguration and service product iteration, the users’ personalized requirements are stimulated. Personalization represents the uniqueness of their actual or perceived use (Fang, 2019). In e-commerce, personalized services affect consumer loyalty (Zhang et al., 2011). In the online learning environment, the personalized value of learners is inevitable to measure the value learners can perceive. Therefore, we define the personalized value as the fourth dimension of the platform value construct. Building on prior research in user-perceived value, we measure the value construct includes emotional, social, functional, and personalized factors. Personalized value refers to the specific needs of an individual user that can obtain from the service provider, who is familiar with user preferences and can/may provide more personalized services.

**Table 1:** Definition of value co-creation

ID	References	Platform value	Definition
1	Mostafa (2015)	Functional value	Functional value refers to the extent to which the service can achieve its utilitarian goals
2	Walsh et al. (2014)		Functional value refers to the practical or technical benefits that consumers can obtain by using a product
3	Mostafa (2015)	Emotional value	Emotional value refers to the various affective states may occur as a result of a consumption experience
4	Walsh et al. (2014)		Emotional value refers to the mental or psychological needs of consumers and the utility they derive from the feelings or affective states that a product generates
5	Mostafa (2015)	Social value	Social value refers to the benefits resulting from the interface between the service provider and other users within the service context
6	Walsh et al. (2014)		Social value refers to the social utility that consumption of the product conveys
7	This paper	Personalized value	Personalized value refers to the specific needs of an individual user that can obtain from the service provider, who is familiar with user preferences and can/may provide more personalized services

Drawing on the SDL perspective, the current study aims to bridge the gap between the growth potential of learning platforms' performance and the limited understanding of what platform value is.

### Structure of learner engagement

Recently, scholars have gradually focused on the study of the structure of learner engagement. For instance, according to Dȩbiec (2017) learners' resistance to active engagement leads to high discontinuation rates in online learning. Farrel and Brunton (2020) explored the structural and psychological factors that affect online learner engagement. Engagement is a key locus for interventions to reduce dropout rates, increase learning achievement, and help learners to develop the skills that are essential to compete for the jobs of the future. However, online learning is lower than face-to-face learning in terms of learning engagement, which is a significant antecedent for good learning outcomes. Keeping users enrolled and engaged is a challenging task as a personal touch by the instructor is missing or limited (Zhang et al., 2017). Table 2 shows the engagement dimensions from the mainstream scholars.

**Table 2:** Summary of learner engagement dimensions

References	Engagement dimensions
Fredericks et al. (2004)	Behavioral, emotional, cognitive and studying engagement
Henrie et al. (2015)	Motivation, participation, academic achievement, and interaction with classmates or instructors
Joksimović et al. (2017)	Behavioral, academic, cognitive, and affective engagement
Chen (2018)	Cognitive dimension (thoughts), emotional dimension (feelings), and behavioral dimension (action or interaction)
Ding et al. (2018)	Behavioral, cognitive, and emotional engagement
Dent et al. (2020)	Behavioral, cognitive, emotional, and social engagement

In this study, we treat academic engagement as instrumental outcomes similar to Joksimović et al. (2017) but we treat learner engagement as a process affecting learning outcomes. Learner engagement describes a learning task or a value referring to the cognitive process, active participation, and emotional involvement of learners in specific learning procedures. Therefore, we define learner engagement as a 3-dimension construct: behavioral, cognitive, and emotional engagement.

Behavioral engagement is related to learner participation, such as time spent on learning activities. Emotional engagement draws on the idea of learners' affective reactions to learning and learning environments (Fredericks et al., 2004). Emotional engagement may include self-reporting or through visible positive emotion expressions (Ding et al., 2018). Cognitive engagement refers to learners' psychological and cognitive involvement in learning activities (Fredericks et al., 2004). Cognitive engagement as the psychological component encompasses learners’ willingness to expend extra effort on learning. In general, active collaborative learning and engagement influence learning performance positively and significantly (Blasco-Arcas et al., 2013).

### Learning outcomes

With the rapid development of online learning, attention to online learning outcomes has increased. The ultimate goal of increasing learner engagement is to promote online learning performance. However, the increase in learner engagement does not always guarantee a high-quality learning experience or academic performance (Joo et al., 2013). Thus, the process from learner engagement to learning outcomes still needs more specific research. To this end, Deng and Benckendoff (2021) presented some propositions to promote the learning experience in online learning. In order to promote student engagement, teachers actively become learners to participate in online learning and continuously train themselves (Moreira et al., 2021). Thus, Moreira et al. (2021) emphasized teachers’ active learning and proposed a model to guide teachers training within Digital Transformation. Unlike this paper, they studied the relationship between learner engagement and learning outcomes from the teacher's perspective. Learners report that sometimes their learning intensity is high but rarely fun; although sometimes they report the opposite: positive emotional response, but the low academic intensity and low academic performance (Fredericks et al., 2004). Learners have more than one indicator of their learning outcomes, but more attention has focused on academic achievement. The learning outcome is the measure of the effectiveness of a learning platform (Zhang et al., 2017). Research links behavioral, cognitive, and emotional engagement to learning outcomes such as learner persistence and academic achievement. In China, before the emergence of online learning, standardized academic performance test was often the only indicator to measure learning achievement, which was greatly restricted and questioned by researchers and instructors. Thus, we intend to combine instrumental learning outcomes and experiential learning outcomes as learning outcomes evaluation. It was consistent with Yang et al. (2016), who revealed that learning performance consists of both subjective and objective learning outcomes.

Instrumental learning outcomes are an external direction related to standardized testing and formal measurement of academic achievement for online learning, with a focus on the goals that learners plan to achieve. Experiential learning outcomes are the psychological or sociological consequences of individuals (e.g. positive experiences, continuing motivation for long learning outcomes). Experiential learning outcomes emphasize the experiences in the learning process, including satisfaction with the platform, loyalty to the platform, and positive word of mouth. The traditional information technology (IT) platform design is with an overall goal of helping individuals to get jobs done. Thus, more attention comes to the work-related instrumental benefits of platform use, which reflects in information systems research, where perceived usefulness is a primary assessment structure. For example, perceived usefulness has significant effects on students’ continuance intention (Daneji et al., 2019). Today, people are increasingly aware of the value of experiences, such as fulfillment, enjoyment, satisfaction, and meaning. Thus, we identify experiential learning outcomes along with the intended instrumental learning outcomes in the context of online learning as the online learning outcomes.

### Learning platform

**Fig. 1:**
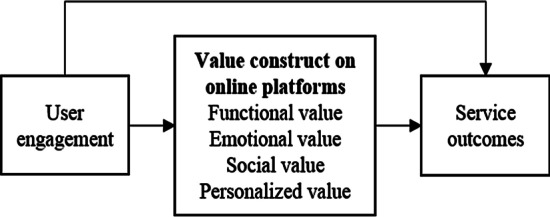
Theoretical model

An engagement platform is defined as physical or virtual touchpoints designed to provide structural support for the exchange and integration of resources, and thereby co-creation of value (Breidbach et al., 2014). Awareness and emotion in online learning platforms are very important to learner engagement (Collazos et al., 2021). Unlike this study, some online guidelines emphasizing awareness and emotional elements are designed by Collazos et al. (2021) to develop online learning platforms better. A digital platform is a way not only for establishing the linkage between information and service, but also engaging different stakeholders for one target. Therefore, the practice of the platform is closely associated with the concept of co-creation (Yu et al., 2019). The motivation of the inherent mechanism to encourage the holistic process and the sustainability of platforms would rely upon the further realization of the value co-creation process. In such a context, this study aims to explain how to evaluate value co-creation in online learning platforms to enhance learning outcomes. This study also proposes a conceptual model that includes user engagement, service outcomes, and the path of value co-creation (see Fig. 1). The mechanism between the input of learner engagement and the output of service outcomes is clarificated for the value co-creation process of the platform.

### Gaps and research questions

Despite several benefits of online learning, retaining learners in online platforms is still challenging (Panigrahi et al., 2018). In academia, previous researches on learners’ continuance usage of technology have paid little attention to specific values learners can pursue. Because the conventional model (e.g., the technology acceptance model (TAM)) holds an assumption that platform users are passive and only respond to technology. The primary factors of technology continuance usage are perceived usefulness and perceived ease of use from TAM (Panigrahi et al., 2018). However, perceived usefulness and perceived ease of use still treat online learners as passive users. The learner engagement is the prerequisite for the value co-creation process but treated as a dependent variable in most literature.

Through a review of the existing literature, we found that several research gaps still exist:

1. Past literature has focused mostly on the passive role of learners, who actually are active users of alternative online platforms. The restrictive view of passive use is not sufficient to clarify users’ continuance usage (Fang, 2019).
2. Although several studies have investigated on the impact of learner engagement on learning outcomes, there is still lack of knowledge about the mechanisms of the value co-creation process to explain how learner engagement turns into learning outcomes. For instance, past research has revealed that stimulating learner engagement plays a crucial role in achieving better learning outcomes (Zhang et al., 2017). We use value co-creation theory for exploring the mechanism of user engagement affecting service outcomes.
3. The platform value assessment is not suitable for the characteristics of online interactive platforms, especially in the era of the experience economy. Value co-creation is difficult to observe empirically, whereas user engagement is observable and thus more likely to be design-able and manageable (Prahalad & Ramaswamy, 2004). An in-depth exploration of learner engagement in value co-creation process is crucial to provide insightful perspectives for improving learning outcomes and users’ continuance usage in less restrictive online learning platforms.

Thus, according to the above research gaps, the primary research questions of this paper are posed:

1. Whether learner engagement promotes value co-creation in an online learning platform? And if so, how does value co-creation affect learning outcomes, especially the instrumental learning outcomes and experiential learning outcomes?
2. How to improve learning outcomes and learners’ continuance usage of the platform compared to other platforms through learner engagement and the value co-creation process in an online learning setting?

## Conceptual model and hypotheses

**Fig. 2:**
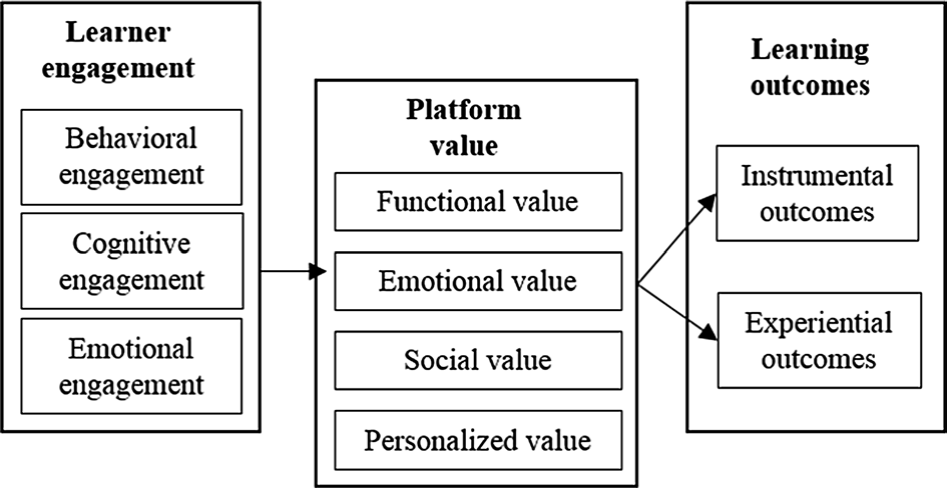
Conceptual model of learner value co-creation process

It is hoped that innovation in online learning platform can lead to greater learner engagement through continuance commitment, normative commitment and affective commitment (Meyer & Allen, 1991), which are taken into account and used as a reference by the proposed model. In our research model, learner engagement is a multidimensional construct consisting of behavioral, cognitive, and emotional sub-types. Researches have linked behavioral, cognitive, and emotional engagement to learning outcomes, such as achieving desirable academic and social outcomes and even feeling emotionally connected. For example, when learners engage in understanding and participation, they are more prone to show interest in learning and to feel emotionally connected (Yang et al., 2019). In this study, the course design and interaction design are standardized on the same platform because we focus on the value co-creation process from the perspective of learners. We conducted a case study from the same online learning platform (an English learning App platform). The lens of SDL paves a promising way to co-create user-perceived value because it presents a comprehensive view of the relationship between learner engagement and platform learning outcomes. We examine the relationship between learner engagement and learning outcomes and the mediating impact of platform value on the relationship. We assume that as learners become more engaged in the online learning process, perceived value increases and learning outcomes improve. The platform value construct consists of functional value, emotional value, social value, and personalized value because we find that personalized value is imperative in online service. A core value in use proposition is that value is created over time through a user's cognitive and experiential interactions with a service provider (Sandström et al, 2008). Therefore, we associate all three types of engagement (behavioral, cognitive, and emotional) with user-perceived value, which is platform value in the model (see Fig. 2).

### Behavioral engagement

Behavioral engagement includes completing work and adhering to rules (Fredericks et al., 2004). Behavioral engagement includes learner effort, persistence, participation, and adherence to the structure, which is critical for achieving positive academic outcomes and preventing dropouts. Behavioral engagement in online learning activities is positively correlated with curriculum performance (Tsay et al., 2018). In general, there is ample evidence that behavioral measurement is associated with academic achievement and success (Fredericks et al., 2004). A high level of behavior engagement embodies better value co-creation, suggesting better instrumental learning outcomes and experiential learning outcomes in online learning. We also expect that high learner behavioral engagement will increase learning outcomes through our four dimensions of platform value. We propose the following hypotheses:

#### H1a

Behavioral engagement improves functional value.

#### H1b

Behavioral engagement improves social value.

#### H1c

Behavioral engagement improves emotional value.

#### H1d

Behavioral engagement improves personalized value.

#### H1e

The impact of behavioral engagement on instrumental learning outcomes is mediated by platform value.

#### H1f

The impact of behavioral engagement on experiential learning outcomes is mediated by platform value.

### Cognitive engagement

Cognitive engagement refers to learners' perceptions of themselves and learning. Cognitive engagement includes motivation, effort, and strategy use (Fredericks et al., 2004). Cognitive engagement leverages the concept of investment and combines thoughtfulness and willingness to understand complex ideas. It also means mastering difficult skills using self-regulating meta-cognitive strategies, such as planning, monitoring, and assessing a person's understanding of a topic or a task. Deep cognitive engagement is facilitated by working with domain-specific tools (Shernoff, 2013). Without intrinsic motivation, the learning outcomes are hard to be as well as expected. Literature measured cognitive engagement as an intrinsic motivation for learning. Thus, we expect cognitive engagement to influence instrumental learning outcomes and experiential learning outcomes. We also propose that cognitive engagement impacts online learning outcomes through our four learner-related dimensions of platform value. We propose the following hypotheses:

#### H2a

Cognitive engagement improves functional value.

#### H2b

Cognitive engagement improves social value.

#### H2c

Cognitive engagement improves emotional value.

#### H2d

Cognitive engagement improves personalized value.

#### H2e

The impact of cognitive engagement on instrumental learning outcomes is mediated by platform value.

#### H2f

The impact of cognitive engagement on experiential learning outcomes is mediated by platform value.

### Emotional engagement

Emotional engagement as defined by Allen and Meyer (1990) means that employee's attachment, identification and participation in the organization. In a learning situation, emotional engagement refers to the positive emotions of learners in learning activities, including interest, boredom, happiness, sadness, and anxiety (Fredericks et al., 2004). Emotional engagement in a non-formal online learning setting includes interests, values, emotions, and emotional attitudes toward online learning. Although learners' emotional responses to online learning are critical in learning, research linking emotional engagement to achievement is limited (Reid et al., 2016). The unique contribution of emotional engagement to learning outcomes has more space to research on. Learners' emotional engagement has a positive influence on active learning, which builds upon the results of previous studies that make general reference to the effect of engagement without explicitly measuring the emotional dimension (Blasco-Arcas et al., 2013). The more positive of emotional engagement, the greater will be the possibility of generating active learning (Molinillo et al., 2018). The emotional engagement has more to do with the pleasant and unpleasant emotions learners feel. Thus, we propose that emotional engagement influences learning outcomes, and emotional engagement impacts learning outcomes through our four dimensions of platform value. We propose the following hypotheses:

#### H3a

Emotional engagement improves functional value.

#### H3b

Emotional engagement improves social value.

#### H3c

Emotional engagement improves emotional value.

#### H3d

Emotional engagement improves personalized value.

#### H3e

The impact of emotional engagement on instrumental learning outcomes is mediated by platform value.

#### H3f

The impact of emotional engagement on experiential learning outcomes is mediated by platform value.

We incorporate value co-creation theory into the online service (online learning) and assume that user engagement, which is learner engagement, impacts the value creation process and service performance (learning outcomes). Our assumptions are also consistent with that the importance of user interactions that co-create value should be increased (Ostrom et al., 2015), and the interactions are mainly user engagements (Roy et al., 2018).

## Methodology and data analysis

We first describe how to measure our constructs and how to collect the data from the survey. Then we test the reliability and validity of the measurement.

### Selection of measurements

Our research conducted in the same online learning platform. A questionnaire was employed to examine our model. The questionnaire is divided into five sections to specifically address the research questions formulated in this study (see Appendix). Our research constructs were measured using validated items from prior studies. Section one is used for identifying learner engagement, including behavior engagement, emotional engagement, and cognitive engagement. The question items of the learner engagement construct were adapted from Skinner et al. (2009) and the online learning experience scale (Deshwal et al., 2017). Section two is used for identifying values, including functional value, social value, emotional value, and personalized value. The functional, emotional and social value were measured using items adapted from Walsh et al. (2014) and Mostafa (2015). Personalized value was adapted from Parasuraman et al. (1988) and Xu et al. (2009). Section three consists of experiential outcomes and instrumental outcomes. Experiential outcomes were measured using items adapted from Alavi (1994) and Alavi et al. (2002). Instrumental outcomes were measured using items adapted from Venkatesh et al. (2003). Section four consists of four items for the online learning platform measurement. The platform was measured using items adapted from Byrd and Turner (2000). These items were slightly modified to fit the context of the current study (online learning). Section five of the control variables include eight questions for capturing the respondents' demographic information, level of education and their experiences of online learning. Five-point Likert scales ranging from 1: strongly agree to 5: strongly disagree was used.

### Survey deployment and data collection

All the constructs in the study were measured using multiple scales. The items were adapted from previous validated research instruments with wording modification where necessary to tailor the scales to the current study context and avoid response bias. We first presented our instrument to experts from practice and academia for evaluation. Then we conducted a pilot survey to get feedback on the usability of our instrument. The pilot includes 50 respondents who received a small cash reward by We-chat, which is the most popular communication software in China. Upon completion, we asked respondents to give open feedback on the comprehensibility of the measures and any other issues they may have faced in completing the instrument. We screened the data to eliminate incomplete responses and monitor the time each respondent spends on the instrument by the online system itself. Based on users’ feedback on item clarity, we modified the items that were hard-to-understand as well as making sure the meaning of the construct remains the same. Finally, the target population for this study consisted of 248 individuals from the users of the same online learning platform by We-chat excluding the initial 50 respondents. For the effect of our study, we also excluded users who did not log in to the online learning platform within a week. We also controlled the model for learners’ characteristics such as age, gender, level of education. Eventually, we got 200 complete responses for this study. The sample was split evenly across the genders (53% male), and the average age of the respondents was 26 years (SD = 7). All of the respondents had completed high school education and all of them use computers or smart-phones every day. We assume that learner engagement impacts the value co-creation process and learning outcomes. However, this relationship is contingent on the quality of online learning interactive platforms. IT platform plays a significant role in enabling firms to offer superior services and ultimately build stronger relationships with their customers (Zhang et al., 2011). To focus on the core dependent and mediating variables, we surveyed the same English learning App platform. We measured users' subjective evaluation of the platform by using the scale of Byrd and Turner (2000).

### Data analysis

A Partial Least Squares (PLS) structural equation model is composed of two sub-models: a measurement (outer) model and a structural (inner) model. The measurement model represents the relationships between the observed data and the constructs, whereas the structural model represents relationships between constructs.

To assess the measurement properties of instruments, we first examined the reliability and validity of first-order reflective constructs. Second, we tested whether the personalized value as a first-order construct exists. Finally, we evaluated the validity of platform value, which conceptualized as a second-order construct with reflective indicators. To measure the proposed constructs, we adapted all items from previous validated research instruments and only made some rewording to fit the context of this study. Prior to the data collection, we ensured the content validity and face validity of the items and constructs by pretesting the questionnaire to 50 respondents as well as experts from practice and academia, and incorporated their feedback in the design of the final questionnaire.

We performed Exploratory factor analysis to check if the proposed factors are consistent with our data. We tested the reliability of each construct using Cronbach’s alpha and Fornell’s composite reliability, and all reliability of constructs had a value higher than the minimum cutoff score of 0.7 (Chin, 1988; Wang, 2019). The indicator reliability was higher than the minimum threshold of 0.5. To assess the convergent validity, we calculated the individual indicator loading (the mini-mun threshold for indicator loading is 0.7) and average variance extracted (AVE) to evaluate the adequacy of measurement models, demonstrating satisfactory explanatory power to the measurement models of the key model constructs. AVE values for all constructs, ranging from 0.639 to 0.758, were above 0.5 (see Table 3). These findings demonstrated adequate reliability and convergent validity of the measurement models (Bagozzi & Yi, 1988; Chin, 1988).

**Table 3:** Reliability and Validity of measurement scales (n = 200)

Constructs	Items	Loadings	T-value	CR	AVE	CA	rho-A
Behavioral engagement (BE)	BE1	0.879	0.401	0.916	0.732	0.878	0.879
	BE2	0.913	0.327				
	BE3	0.941	0.371				
Cognitive engagement (CE)	CE1	0.795	0.413	0.898	0.639	0.859	0.862
	CE2	0.839	0.463				
	CE3	0.807	0.351				
Emotional engagement (EE)	EE1	0.855	0.330	0.901	0.740	0.883	0.883
	EE2	0.886	0.302				
	EE3	0.810	0.277				
	EE4	0.780	0.289				
Functional value (FV)	FV1	0.750	0.339	0.836	0.673	0.737	0.762
	FV2	0.862	0.454				
	FV3	0.870	0.419				
Emotional value (EV)	EV1	0.867	0.351	0.908	0.711	0.864	0.867
	EV2	0.749	0.298				
	EV3	0.780	0.303				
	EV4	0.812	0.291				
Social value (SV)	SV1	0.860	0.407	0.932	0.732	0.908	0.909
	SV2	0.869	0.398				
	SV3	0.768	0.396				
Personalized vale (PV)	PV1	0.859	0.282	0.924	0.753	0.868	0.890
	PV2	0.855	0.277				
	PV3	0.864	0.290				
	PV4	0.892	0.302				
Instrumental outcomes (IO)	IO1	0.771	0.177	0.937	0.714	0.845	0.888
	IO2	0.830	0.188				
	IO3	0.904	0.205				
	IO4	0.852	0.192				
	IO5	0.840	0.206				
	IO6	0.867	0.214				
Experiential outcomes (EO)	EO1	0.859	0.314	0.911	0.758	0.849	0.890
	EO2	0.855	0.306				
	EO3	0.864	0.280				
	EO4	0.892	0.277				

^*^See the Appendix for detailed questions corresponding to each indicator item

For discriminant validity, the square root of the AVE should exceed the variance shared between a construct and other constructs (Chin, 1998). Table 4 shows the square roots of the AVE values were consistently greater than all corresponding correlations. Therefore, confirmatory factor analysis results provide evidence of high internal consistency as well as convergent and discriminant validity.

**Table 4:** Interconnections of the latent variables for first-order constructs

Constructs	BE	CE	EE	EV	FV	PV	SV	IO	EO
BE	***0.856***								
CE	0.822	***0.799***							
EE	0.833	0.711	***0.860***						
EV	0.791	0.749	0.730	***0.843***					
FV	0.761	0.769	0.729	0.772	***0.751***				
PV	0.741	0.710	0.480	0.509	0.502	***0.867***			
SV	0.769	0.726	0.718	0.822	0.735	0.499	***0.856***		
IO	0.741	0.436	0.456	0.522	0.490	0.855	0.496	***0.843***	
EO	0.776	0.496	0.479	0.514	0.482	0.819	0.480	0.802	***0.847***

^*^Diagonal items in italics show the square root of the AVE. Off-diagonal items show the correlations between constructs

The reliability of the scale is assessed using internal consistency measures assume equal weighting of items. Convergent validity and discriminant validity were confirmed from Table 3 and Table 4. The measurement model was evaluated using the criteria of Wang (2019) indicating empirical support for the structure of composites and that our measures have good measurement properties.

### Platform value: a second-order construct

As conceptualized above, platform value is a multidimensional construct, which represents the comprehensive measure of the level of platform value along the four dimensions of functional value, emotional value, social value, and personalized value. We developed separate scales to uniquely measure each construct. Next, we test the impact of each of these constructs on the second-order construct of platform value. The individual dimensions of platform value should not be considered in isolation from each other but should be treated in a collective and mutually reinforcing manner.

We established a higher-order latent variable using the repeated indicators of the first-order latent variable. The four first-order factors in the model (FV, EV, SV, PV) have significant statistical significance and high correlation, and the positive correlation between the four first-order factors indicates that a high value on one of these factors did not rule out the possibility of high values on other factors (Zhu, 2004). Thus, there is a possibility of a higher-order factor structure. The correlation between these four first-order factors is below the 0.90 cutoff value (Bagozzi et al., 1991), showing acceptable discriminant validity. All the standard loading is significant and greater than 0.7, indicating evidence of good convergent validity. The composite reliability was estimated from path loading to be 0.961 (see Table 5), indicating high reliability for the second-order construct (Vargo & Lusch, 2008). Therefore, on both theoretical and empirical grounds, the conceptualization of platform value as a higher-order, multidimensional construct is justified. The platform value as a second-order construct reflects the phenomenological nature of the interaction between users and service providers.

**Table 5:** Estimation of the second-order construct of platform value

Second-order construct	First-order constructs	Standard loadings	t-statistics	Composite reliability
Platform value	Functional value	0.770	20.139	0.961
	Emotional value	0.892	38.459	
	Social value	0.874	33.692	
	Personalized value	0.891	38.402	

A number of procedures were taken to diminish the threat of common method bias. First, we conducted the Harmon one factor test (Podsakoff et al., 2003), which required that we loaded items used to measure all the constructs in the model into a single exploratory factor analysis. As more than one factor was extracted and less than 50 percent of the variance can be attributed to the first factor, common method bias was unlikely to be a significant problem with our data (Keil et al., 2007). Thus, Harman’s single-factor test indicated that no general factor that accounted for the majority of the covariance among all the factors existed. Second, common method bias exists if correlations are higher than 0.9 (Pavlou et al., 2007). The highest correlation coefficient in the correlation matrix was 0.855. Third, the model fit index standardized root mean square residual (SRMR) is adopted. A model with an SRMR less than 0.08 is acceptable (Henseler et al., 2016). Hence, the overall model is justified.

### Model estimation and implementation

The variance-based partial least squares (PLS) method was chosen for the data analysis because: (1) We could not guarantee that most of the data followed a normal distribution. (2) PLS is prediction-oriented and recommended for early stages of theory development (Fornell & Bookstein, 1982). Given that there has been little prior theory and few empirical studies on online learning from the perspective of the SDL, this topic is novel. (3) PLS path modeling is a preferred statistical tool for the structural equation modeling method that uses a component-based approach to estimation. Our PLS model includes platform value, three antecedent factors of learner engagement, as well as experiential outcomes and instrumental outcomes as the dependent variables.

**Fig. 3:**
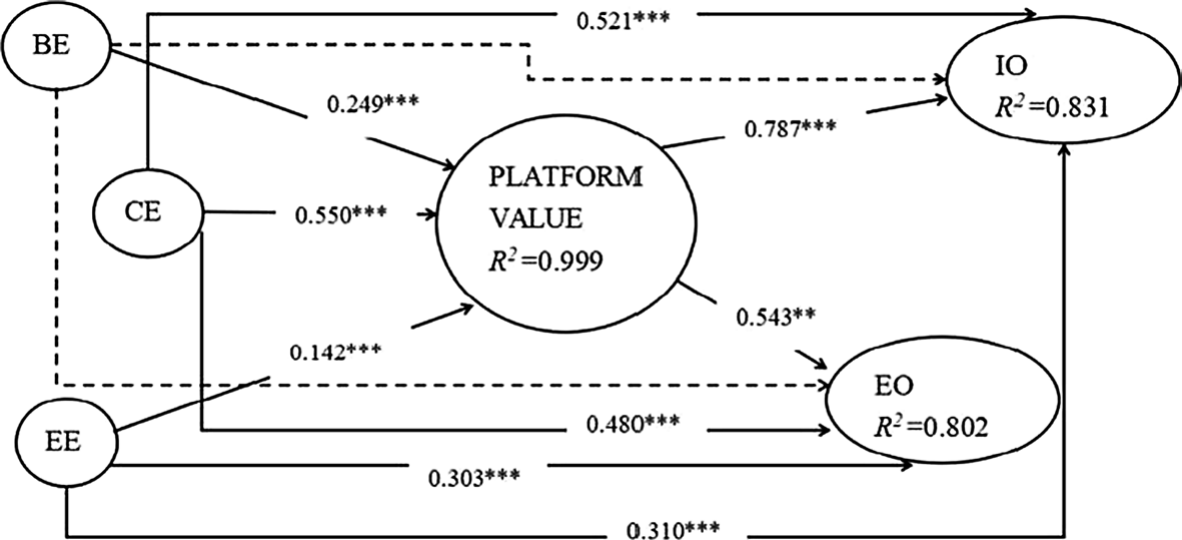
Structural model analysis. **p < 0.05; ***p < 0.01

To evaluate the structural model, we tested the significance of the relationships between the constructs. We applied a bootstrap procedure with 500 sub-samples to determine the significance of path estimates and to compare path estimates statistically. In order to test if value co-creation theory provides a satisfactory explanation of the mediation model, we followed the approach described by Baron and Kenny (1986). Mediating effects are important because they allow researchers to isolate the mechanisms underlying observed correlations between exogenous factors and dependent variables. First, we estimated the partial impact of learner engagement on learning outcomes without the presence of the mediator. The regression coefficients of BE, CE and EE were positive and significant for the learning outcomes, which showed that learner engagement had a positive impact on learning outcomes. Next, we estimated the full model, coefficients of direct paths from BE, CE, and EE to the experiential outcomes and instrumental outcomes were smaller. All the results were demonstrated in Fig. 3. The effect from BE to IO and EO mediated by platform value was not statistically significant anymore, indicating that behavioral engagement impacts learning outcomes fully mediated by platform value. The effect from CE and EE to IO and EO mediated by platform value was smaller than the direct effect, indicating that cognitive engagement and emotional engagement impact learning outcomes partially mediated by platform value. The mediator mechanism of platform value was verified.

All the results show that the structural model in this paper has good explanatory power and predictive power among the associated variables. As shown in Fig. 3, R^2^ values range from 0.802 to 0.999 idicating a reasonably good fit of the overall model. According to the effect sizes defined by R^2^ values according to Cohen (1988), these effects can be classified as large ones. Hair et al. (2011) also suggested that the values of 0.75, 0.5 and 0.25 for R^2^ indicate respectively that the model is substantial, moderate and weak. The partial mediation model demonstrates high explanatory power, as shown in Fig. 3. The model explained 83.1% of the variance in instrumental outcomes and 80.2% of the variance in experiential outcomes.

We summarized the results of our hypotheses in Table 6. We confirm personalized value by our study, which impacts learning outcomes significantly. This is consistent with Hoić-Božić et al. (2016) argument that those learners who performed personalized collaborative e-learning activities achieved better course result. Our analysis shows that behavior engagement, cognitive engagement, and emotional engagement demonstrate the impact on learning outcomes through the mediating effect of platform value. Further, through the mediation of platform value, we verified a significant path from learner engagement to both instrumental and experiential outcomes.

**Table 6:** Summary findings of hypotheses tests

	Hypotheses	loadings	T-value	Finding
H1a	BE → FV	0.258**	2.787	Supported
H1b	BE → SV	0.310***	3.733	Supported
H1c	BE → EV	0.250***	2.839	Supported
H1d	BE → PV	0.102	0.924	Not Supported
H1e	BE → PV → IO	0.196***	2.968	Supported
H1f	BE → PV → EO	0.135***	2.575	Supported
H2a	CE → FV	0.564***	6.169	Supported
H2b	CE → SV	0.500***	5.472	Supported
H2c	CE → EV	0.474***	5.117	Supported
H2d	CE → PV	0.528***	5.308	Supported
H2e	CE → PV → IO	0.433***	6.184	Supported
H2f	CE → PV → EO	0.299***	5.429	Supported
H3a	EE → FV	0.046	0.527	Not Supported
H3b	EE → SV	0.056	0.856	Not Supported
H3c	EE → EV	0.179***	2.470	Supported
H3d	EE → PV	0.244***	2.725	Supported
H3e	EE → PV → IO	0.112***	2.493	Supported
H3f	EE → PV → EO	0.077**	2.164	Supported

^**^p < 0.05; ***p < 0.01

## Discussion

The objective of this study was to explain how to improve online learning outcomes and users’ continuance usage, which maintains e-commerce sustainability through learner engagement and the value co-creation process in an online learning setting. The findings of this study are as follows: (1) This study expands the extant literature by explaining how the value co-creation process influences learning outcomes as an antecedent in online learning environment. The users achieve specific values through their engagement positively impacting on the learning outcomes, which, in turn, have contributed to the sustainability and improved intention for more user engagement. (2) The service nature of online learning suggests that research on the factors that lead to sustainability of platforms should consider users’ perceived value as one of the prominent determinants of service outcomes and continuance usage. This observation suggests the necessity to incorporate the service-dominant logic as the theoretical base in the current study. (3) The combination of value co-creation theory and online learning is a new method for learning outcomes enhancement and online platforms continuance usage. The values learners achieve are the fundamental reason for them to stick on this learning platform comparing other similar learning platforms. Under such a circumstance, the perceived value from the platform is the fundamental difference from other platforms.

These findings make several contributions to the research and practice around learner engagement and learning outcomes in the context of non-formal online learning. From the perspective of service science and its core concepts of value co-creation, promoting user engagement is critical to service outcomes and online service performance. The positive correlation between learner engagement and learning outcomes through the value co-creation process found in this study supports previous studies (Zhang et al., 2017).

All the hypothesized relationships in the proposed model were found to be significant expect the 3 hypotheses as following: BE → PV; EE → EV; EE → SV.

H1d BE → PV is not supported. To our best knowledge, we didn’t find any literature to support or against this path from behavior engagement to personalized value. A possible explanation for the rejection is that: (1) We defined the personalized value in the context of online learning platforms. (2) The observation derived from users’ behavioral engagement from the survey is not comprehensive enough to support the functionality of sound personalized services.

In terms of emotional engagement, items that tap behavioral engagement and emotional engagement are often combined in a single scale. This practice makes it more difficult to identify the precursors and consequences of each type of engagement (Frrdricks et al., 2004). Behavioral engagement is more observable. However, emotional engagement is an internal state that provides the impetus to engage certain academic behaviors (Finn & Zimmer, 2012), which is difficult to measure to some extent. According to Skinner and Pitzer (2012), “emotion is likely the fuel for the kind of behavioral and cognitive engagement that leads to high-quality learning.” Thus, it is hard to recognize the emotional engagement from other kinds of engagement to some extent, which integrates prior research that among the different types of engagements, behavior engagement is relatively easier to measure and collect (Wang, 2017). One possible explanation for the rejection of emotional engagement is that there was not enough information in the survey to distinguish between emotional engagement and other engagements. The evidence also suggests a need to develop and use multiple approaches to measuring engagement in academic work rather than rely only on self-report instruments (Greene, 2015).

### Theoretical implications

Contemporary research in online learning almost unequivocally argues for failing to investigate factors that contribute to online learning (Evans et al., 2016). Our research contributes to online learning research from the perspective of service science and its core concept of value co-creation.

First, our results indicate strong support for our main assertion that learner engagement plays a significant role in enhancing learning outcomes and platform value. The results echo the previous research that customer engagement has a direct and positive effect on customer value creation (Zhang et al., 2017). Platform value mediates the learner engagement on learning outcomes after controlling the characteristics of individuals and interactive platforms.

Second, this study enriches value co-creation research from user experiences, and we define that the platform value is the perceived value with unique user experiences. Previously, most studies have identified 3 dimensions of value measurement. Although many works of literature have shown the importance of personalized value for service performance, its impact is still at the early stage in the context of online learning value co-creation process. The process of creating value in most research of online context is rapidly shifting from a company-centered perspective to a personalized customer experience perspective. Thus, the second-order construct platform value is suitable for the characteristics of online interactive platforms, especially in the era of the experience economy. This study also demonstrates the mediating role of platform value in bridging the influence of user engagement on service outcomes.

Third, previous research has focused on customer engagement in value co-creation, but limited research treats learners as customers or users. For online learning business, learners are users. We actively contribute to the development of the literature for emerging online learning values by developing and empirically validating learner engagement in influencing online learning outcomes. Our research suggests that learner engagement has a significant indirect and direct effect on learning outcomes.

Fourth, we use both instrumental learning outcomes and experiential learning outcomes to measure online learning outcomes. Learning outcomes constitute both instrumental learning outcomes and experiential learning outcomes, which can evaluate online learning outcomes for future research, especially in the era of the experience economy.

As regard to behavioral engagement, our results are in line with that high behavioral engagement is related to higher levels of achievement (Shernoff, 2013). Our results match the prior research that has demonstrated a consistent positive relationship between behavioral engagement and achievement (Li & Baker, 2018). Our results reveal that behavioral engagement improves both instrumental and experiential learning outcomes, which is not consistent with the conclusion that high behavioral engagement is negatively associated with learners’ well-being (Fredricks et al., 2004). About cognitive engagement, our results suggest that cognitive engagement influences instrumental learning outcomes and experiential learning outcomes through the mediation of platform value, which is consistent with that the link between cognitive engagement and achievement is complex and not unidirectional (Li & Baker, 2018). In terms of emotional engagement, as we observed before, there is limited discussion about the influence of emotional engagement on learning outcomes. Emotional engagement draws on the idea of users' affective reactions to the service or environments. Thus, it works dramatically for some special context. Our results suggest that emotional engagement influences learning outcomes through the mediation effect of platform value.

### Managerial implications

Value co-creation has been thoroughly conceptualized in the service sector with little empirical research has been done (Currás‐Pérez et al., 2018). First, promoting learner engagement is critical for learning outcomes enhancement. Second, understanding the composition of the platform value construct is necessary to evaluate the learning platforms. Third, for online learning organizations, perceived value is critical for good value creation and good learning outcomes. Fourth, the mediation effect of platform value on the interrelationships between learner engagement and learning outcomes works like a bridge for platforms to stimulate better learners' perceived value and better service outcomes.

Learning materials may be the same, but if learners are fully engaged in the learning process, they build unique user experiences. According to Kaihara et al. (2018), co-creative value equals sustainability for understanding different aspects of value. Since sustainability is at the core of value creation, value co-creation enables service providers to help users promote service performance. Thus, the 4-dimension platform value construct is an effective method for evaluating user engagement outcomes as well as assessing the sustainability of the online platforms. By user engagement outcomes enhancement and sustainability of platforms, online platforms will achieve the goal of "win–win" through the lens of service science and value co-creation. Our study contributes to assessing the commerce sustainability phenomenon from the new perspective of value co-creation theory in the online learning setting.

## Conclusions

This study has made three important theoretical contributions to the literature. First, this study provides a rigorous way to examine a structural model that includes multiple service outcome predictors and provides an integrated model for the value co-creation process of online learning. Through this model, it provides support for the importance of user engagement and platform value as antecedents to service outcomes, as well as the role user engagement plays in promoting platform value. Also, platform value is a second-order construct consisting of functional value, social value, emotional value, and personalized value. Second, this study has not only supported the conceptualization of platform value as a second-order construct with adding the value dimension of personalized value but has also highlighted the role of platform value as the mediator between user engagement and service outcomes. Third, with good instrumental learning outcomes and experiential learning outcomes, the learning platform can promote learner engagement and online learning performance in a sustainable manner. This is the embodiment of a “win–win” value creation process in the platform.

The practical contribution of the article is to provide a new path for promoting online learning outcomes through an empirical study. The study findings contribute to an enhanced understanding of how value co-creation takes place in online service.

### Limitations and future works

This study has certain limitations, and future works can improve these shortcomings. Although our proposed antecedent model was largely supported in the empirical analysis, we must remember that this study is limited by its choice of setting, study design, and choice of variables. We confirmed the impact of learner engagement on value co-creation of online learning, and the validity of the platform value construct, which laid the foundation for the platform value measurement in all the online services for future research. We also note the omission of research variables that could be important in the context of online service. For example, technical aspects of the platform should be considered as variables affecting learning outcomes.

Besides, we did not have the chance to collect the data from the instructor side because we treat the value co-creation process as a learner-oriented process. In the future, the method for collecting users’ engagement will be supported by online big data technology. To this end, in the future, collaborative learning will be taken into account, and the value co-creation process from the dual perspectives of collaborative learning and service-dominant logic will be studied.

Lastly, instructors can improve their teaching and teaching experience by using gestures to stimulate learner engagement and users’ continuance usage (Yang et al., 2020). Therefore, for the sustainability of the learning platform, the tactics of stimulating learner engagement and users’ continuance usage need more serious research.

## Appendix: Measurement items

### Behavioral engagement

**BE1**: I try hard to do well when I use this learning platform comparing other English learning platforms.
**BE2**: I pay more attention when I use this learning platform comparing other English learning platforms.
**BE3**: I focus better when I use this learning platform comparing other English learning platforms.

### Cognitive engagement

**CE1**: I feel important to understand the knowledge from this learning platform.
**CE2**: I am sure I can do an excellent job when I use this learning platform.
**CE3**: I keep working until I finish even when sometimes learning is not that interesting.

### Emotional engagement

**EE1**: I feel good when I use this learning platform.
**EE2**: I feel interested when I use this learning platform.
**EE3**: I feel that learning in this platform is fun.
**EE4**: I get involved when I work on something in this learning platform.

### Functional value

**FV1**: I think that this learning platform has consistent quality.
**FV2**: I think that this learning platform is well made.
**FV3**: I think that this platform has an acceptable standard of quality.

### Social value

**SV1**: Comparing other English learning platforms, I think that social activities on this platform make my studies more interesting.
**SV2**: I think that the social interaction with my lecturers and fellow learners online makes my studies more interesting.
**SV3**: I think that using this platform helps me feel more acceptable among my peers.

### Emotional value

**EV1**: I find my study in this platform interesting.
**EV2**: I prefer using this learning platform to compare other platforms.
**EV3**: I feel relaxed when I use this learning platform.
**EV4**: I feel my progress when I use this learning platform.

### Personalized value

**PV1**: I think this learning platform pays attention to my needs.
**PV2**: I think this learning platform can provide me with adaptive services tailored to my activity context.
**PV3**: I think this platform can provide me with more relevant information tailored to my preferences or personal interests.
**PV4**: I think this platform can provide me with the kind of information or service that I might like.

### Experiential outcomes

**EO1**: Overall I had a great experience using this platform.
**EO2**: My learning experience improves after using this platform.
**EO3**: I would take part in a course similar to the current course of this learning platform given a choice.
**EO4**: I believe I have learned more because of the class format of this learning platform.

### Instrumental outcomes

**IO1**: I think I can improve my grade when I use this learning platform.
**IO2**: I think I learn better when I use this learning platform.
**IO3**: I think I improve my study performance when I use this learning platform.
**IO4**: I think my effectiveness on study is enhanced when I use this learning platform.
**IO5**: I feel easier to do my study then I use this learning platform.
**IO6**: I find this learning platform useful in my study.

### Interactive platform

**IP1**: I think this learning platform offers all the IP features that I need to use.
**IP2**: I think I can get fast-responding IP support when there is a problem concerning IT.
**IP3**: I feel confident that I can get enough IP support when I use this learning platform.
**IP4**: I think this learning platform provides the IP support that other competitors may not have.

### Control Variables

Q39: Gender:

Q40: Age:

Q41: Education:

Q42: How often do you use a computer?

Q43: How do you rate your computer skills?

Q44: How often do you use the Internet?

Q45: Have you ever participated in online learning?

Q46: How often you use an online learning platform?

## Data Availability

The data that support the findings of this research are available on request from the first author.
